# Glycolytic biomarkers predict transformation in patients with follicular lymphoma

**DOI:** 10.1371/journal.pone.0233449

**Published:** 2020-05-22

**Authors:** Ida Monrad, Charlotte Madsen, Kristina Lystlund Lauridsen, Bent Honoré, Trine Lindhardt Plesner, Stephen Hamilton-Dutoit, Francesco d’Amore, Maja Ludvigsen

**Affiliations:** 1 Department of Hematology, Aarhus University Hospital, Aarhus, DK; 2 Department of Pathology, Aarhus University Hospital, Aarhus, DK; 3 Department of Biomedicine, Aarhus University, Aarhus, DK; 4 Department of Pathology, Copenhagen University Hospital, Copenhagen, DK; 5 Department of Clinical Medicine, Aarhus University, Aarhus, DK; Seoul National University College of Pharmacy, REPUBLIC OF KOREA

## Abstract

Follicular lymphoma (FL) is an indolent neoplasia comprising approximately 20% of lymphomas. FL is generally considered incurable, with a median survival exceeding 10 years. A subset of FL patients experiences histological transformation (HT) to a more aggressive lymphoma, resulting in markedly poorer clinical outcome, with a reduced median survival after transformation of 1–2 years. Early, reliable prediction of HT would be valuable in the clinical setting, allowing pre-emptive therapeutic intervention. We previously used proteomics to identify the glycolytic enzymes fructose-bisphosphate aldolase A (aldolase A) and glyceraldehyde-3-phosphate dehydrogenase (GAPDH) as candidate predictors of FL transformation. Now, we use immunohistochemistry to evaluate expression of these enzymes in paired primary FLs from patients with (n = 41) or without subsequent HT (n = 49), to test their value as predictive biomarkers. At initial FL diagnosis, patients with subsequent HT had significantly higher expression of aldolase A and GAPDH (p<0.001 and p<0.01) compared with patients without HT. Furthermore, high expression of aldolase A and GAPDH was associated with significantly shorter transformation free survival (p = 0.018, p = 0.001). These data suggest that high expression of aldolase A and GAPDH, may indicate increased metabolic turnover, and that these enzymes may be useful biomarkers in primary FL for predicting the risk of subsequent lymphoma transformation.

## Introduction

Follicular lymphoma (FL) is an indolent lymphoma derived from germinal center B cells [1]. It is the second most common lymphoid malignancy, accounting for some 20% of all non-Hodgkin lymphomas, and is predominantly a disease of adults, the median age of patients at diagnosis being approximately 60 years [2,3]). FL is generally considered an incurable condition with a median survival time exceeding 10 years in the ‘rituximab era’ [2,4]. The natural history of FL usually follows an indolent course with periods of stable asymptomatic disease not requiring therapeutic intervention, alternating with periods of slow progression, associated with varying degrees of generalized symptoms (B-symptoms), where therapeutic intervention may be considered [2,3]. When treated pharmacologically, FL is generally responsive to treatment, although no curative standard therapy exists [3]. Thus, a portion of patients still experience early progression, treatment refractoriness and histological transformation (HT) to a more aggressive lymphoma subtype, typically diffuse large B-cell lymphoma (DLBCL). The occurrence of HT has a clear adverse impact on the patient’s prognosis, lowering the median survival after transformation being typically reduced to 1–2 years [5,6].

Histologically, FL is characterized by a follicular growth of germinal center B-cells with an admixture of centrocytes and centroblasts and is graded from 1-3A/B depending on the number of centroblasts. Clinically, FL grade 3B, often demonstrate a rather aggressive behavior similar to that of DLBCL and is now recognized as HT if the patient has a previous FL diagnosis [7,8]. HT is seen in up to 45% of patients [4,6]. Given the theory of divergent evolution of transformed FL (tFL) subclones [9], it would be of great clinical value if such subclones could be unequivocally detected early in the course of disease in order to predict the risk of subsequent HT [9,10]. However, the detection of transformed subclones at the time of FL diagnosis is a difficult challenge, requiring the application of sophisticated molecular analyses at the limit of their sensitivity ranges to routine primary diagnostic FL specimens, in order to detect very low frequency tFL subclones, representing a very modest mutational load [9]. Therefore, rather than searching for direct evidence of tFL subclones, we should search for predictors of HT, relying on the expectation that diagnostic FL samples from patients that subsequently experience HT might contain additional biological clues that differentiate them from their non-transforming FL counterparts, based on an acceptance of the premise that divergent evolution of tFL subclones is an event that relates back to the early stages of FL development.

Our group has previously applied this concept, using proteomic assessment of FL diagnostic samples from patients with or without subsequent HT to identify differentially expressed protein profiles [11]. This study was performed in a size-limited cohort of fresh-frozen bulk tumor tissue samples, allowing tumor microenvironmental factors to be included in the assessment. Findings from this proteomic study included the identification of fructose-bisphosphate aldolase A (aldolase A) and glyceraldehyde 3-phosphate dehydrogenase (GAPDH) that, among others, were differentially expressed at the time of FL diagnosis, comparing tumors from patients with or without subsequent HT [11]. Aldolase A and GAPDH are both glycolytic enzymes that contribute to the metabolic turnover of glucose [12–14], which is generally increased in cancer [15]. Overexpression of both these enzymes have been identified in various cancers, and has been associated with poor patient outcomes [16–20]. In addition, GAPDH has been associated with several non-glycolytic cellular regulatory functions, including DNA replication and repair, cytoskeletal organization and apoptosis [13,14].

In the present study, we have examined the predictive potential of the two glycolytic enzymes by immunohistochemical evaluation of their expression in pretherapeutic tumor tissue from time of initial FL diagnosis in patients, with and without subsequent HT. Furthermore, paired samples from the time of HT were included to investigate changes in the expression levels of aldolase A and GAPDH from initial FL diagnosis to the time of overt transformation.

## Patients and methods

### Patients

For this study, a formalin fixed, parraffin-embedded (FFPE) tissue cohort was used with 90 FL patients, diagnosed at the Department of Hematology, Aarhus University Hospital, Denmark, between 1990 and 2015 with FL grade 1-3A. The cohort included 49 FL patients without transformation (non-transformed, nt-FL) during at least 10 years of follow up, and 41 patients with a primary FL diagnosis and subsequently histologically confirmed transformation to DLBCL or FL grade 3B, at least 6 months after primary FL diagnosis (sequentially transformed, s-FL/s-tFL). Lymphoma tissues were routinely prepared using standard procedures. All biopsies were reviewed by an experienced hematopathologist and validated or reclassified according to the 2017 update of the WHO Classification of Tumours of the Haematopoietic and Lymphoid Tissues [7,8]. Clinical data from all patients were included, collected from the Danish Lymphoma Registry (LYFO) [21] and patient records. These data together with immunohistochemical data on vimentin and PAX5 tissue expression in this cohort have previously been published [22].

Two patients were excluded from the cohort during the study because of insufficient tissue availability, thus resulting in cohort reduction for GAPDH analysis (n = 88, nt-FL, n = 49 and s-FL/t-FL, n = 39).

The study is approved by the Regional Ethics Committee of central Denmark Region (1-10-72-276-13) and the Danish Data Agency (1-16-02-407-13). None of the included patients were registered in the Tissue Utilization Register.

### Immunohistochemistry

Four-μm FFPE tissue sections were stained by immunohistochemistry on the Ventana BenchMark Ultra automated slide stainer (Ventana Medical Systems, Roche, Oro Valley, AZ) using standard methods. In brief, sections were deparaffinized using the detergent (EZ-prep, Ventana, Roche, Oro Valley, AZ), heat (72°C) and vortex mixing. Endogenous peroxidase activity was blocked by incubation with 3.0% hydrogen peroxide solution, contained in the OptiView DAB IHC Detection Kit (Ventana, catalog no. 760–700). Heat induced epitope retrieval was applied in order to unmask epitopes, crosslinked as a result of the formalin fixation by heating to 100°C for 32 minutes in acidic buffer. Primary polyclonal rabbit anti-human antibodies against aldolase A (anti-ALDOA) and GAPDH (Product nos. HPA004177 and HPA040067, Atlas Antibodies, Stockholm, Sweden) were diluted (1:200 and 1:1000, respectively) in a Tris buffered diluent with a pH of 7.2, 15 mmol/L NaN3 and protein (Dako catalog no. S202230-2) and incubated for 32 minutes at 36°C. Primary antibody binding was detected with the OptiView DAB IHC Detection Kit (Ventana, catalog no. 760–700) which specifically detects rabbit primary antibodies bound to antigens in the tissue sections. Sections of appendix, tonsil, liver and pancreas were included on each slide as positive/negative controls [23].

### Digital image analysis

Stained slides of whole biopsy sections were scanned at a magnification of x20 using the Hamamatsu Nanozoomer 2.0HT scanner (Hamamatsu, Shizuoka, Japan). Staining quantification was performed using Visiopharm Integrator system 2018.09 (Visiopharm A/S, Hoersholm, Denmark) [22]. All digitalized images of whole biopsy sections were manually reviewed for large areas of distinct non-lymphoid tissue, fatty tissue, tissue folds, artefacts, *etc*. that were not eligible for quantification analysis. These areas were manually excluded, resulting in the definition of the region of interest (ROI), within which the staining quantification analysis could be conducted. Analysis protocol packages (APPs) were designed for both markers by manual image class designation to appropriate tissue areas (*i*.*e*. tissue background, weak-, intermediate- and strong staining intensity) followed by APP training based on tissue sections representing differential biomarker expression levels. Area fractions (AFs) were used as staining quantification outputs, and were defined as the stained area normalized to the ROI. Aldolase A expression levels were based on AFs of strong intensity staining, while GAPDH levels were based on AFs of all staining intensities.

### Statistical analysis

Differences in mean of AFs of FL diagnostic samples from patients with or without subsequent transformation was assessed using Student’s t-test and paired T test. Differences in clinico-pathological features were assessed using Fisher’s exact test and correlation of biomarker expression to clinico-pathological features was evaluated using Spearman rank test. Time related endpoints were analyzed using the Kaplan Meier method, with transformation-free survival (TFS), progression-free survival (PFS) and overall survival (OS) as endpoints. TFS was defined as the time from the initial FL diagnosis to the date of histologically confirmed HT or censoring. PFS was defined as the time from the initial FL diagnosis to the date of progression of disease or censoring. OS was defined as the time from the FL diagnosis to the date of death by any cause or censoring. Cutoffs for high versus low expression levels for the survival analysis were based on the 75^th^ percentile of expression. P-values below 0.05 were considered significant. Statistical analyses were performed in STATA version 15.1 (StataCorp).

## Results

The patient cohort comprised a total 90 patients, including 46 males and 44 females (Table 1). The age at diagnosis ranged from 25 to 83 years with a median of 57 years. FL patients with subsequent transformation (s-FL, n = 49) had a more adverse risk profile compared with patients without HT (nt-FL, n = 41), with a more advanced Ann Arbor stage, higher FLIPI score, typically with LDH-elevation, and bone marrow involvement. Aldolase A analysis was conducted on the full patient cohort of 90 patients, while insufficient tissue availability resulted in exclusion of 2 patients for GAPDH analysis, (n = 88; nt-FL, n = 49 and s-FL/t-FL, n = 39). The cohort reduction did not affect the cohort characteristics.

**Table 1:** Clinico-pathological features.

Characteristics	All n = 90 n (%)	nt-FL n = 49 n (%)	s-FL n = 41 n (%)	P-value
Sex				NS
Male	46 (51)	21 (43)	25 (61)	
Female	44 (49)	28 (57)	16 (39)	
Age at FL diagnosis				NS
Median	57	57	57	
Range	25–83	35–83	25–78	
Ann Arbor stage				<0.001
I-II	29 (32)	24(49)	5 (12)	
III-IV	59 (66)	24 (49)	35 (85)	
Unknown	2 (2)	1 (2)	1 (3)	
FLIPI				0.001
Low	33 (37)	25 (51)	8 (20)	
Intermediate	28 (31)	16 (33)	12 (29)	
High	25 (28)	6 (12)	19 (46)	
Unknown	4 (4)	2 (4)	2 (5)	
LDH-elevation				0.038
Yes	15 (17)	4 (8)	11 (27)	
No	71 (79)	43 (88)	28 (68)	
Unknown	4 (4)	2 (4)	2 (5)	
B-symptoms				NS
Yes	22 (24)	9 (18)	13 (32)	
No	63 (70)	38 (78)	25 (61)	
Unknown	5 (6)	2 (4)	3 (7)	
Performance score				NS
< 2	85 (95)	46 (94)	39 (95)	
≥ 2	2 (2)	2 (4)	0 (0)	
Unknown	3 (3)	1 (2)	2 (5)	
Bone marrow				0.035
Involvement	26 (29)	9 (18)	17 (41)	
No	50 (56)	33 (67)	17 (41)	
Unknown	14 (15)	7 (14)	7 (18)	
Anemia				NS
Yes	6 (7)	1 (2)	5 (12)	
No	81 (90)	47 (96)	34 (83)	
Unknown	3 (3)	1 (2)	2 (5)	
FL histology				NS
FL NOS	1 (1)	0 (0)	1 (2)	
FL grade 1–2	74 (82)	39 (80)	35 (86)	
FL grade 3A	15 (17)	10 (20)	5 (12)	
Initial treatment				NA
Alkylator-based	24 (27)	16 (33)	8 (20)	
Antracyclin-based	8 (9)	2 (4)	6 (15)	
Chlorambucil	20 (22)	11 (22)	9 (22)	
Rituximab only	9 (10)	2 (4)	7 (17)	
Radiation only	7 (8)	5 (10)	2 (5)	
Watch and wait	20 (22)	13 (27)	7 (17)	
Other	2 (2)	0 (0)	2 (5)	
R-Chemo	29 (32)	11 (22)	18 (44)	
Aldolase A expression				<0.001
Low	67 (74)	44 (90)	23 (56)	
High	23 (26)	5 (10)	18 (44)	
GAPDH expression				<0.001
Low	66 (75)	44 (90)	22 (56)	
High	22 (25)	5 (10)	17 (44)	

Immunohistochemical staining of Aldolase A and GAPDH exhibited similar staining profiles with diffusely stained cytoplasm of both neoplastic and non-neoplastic cells of the tumor microenvironment. Additionally, some nuclei were positively stained for GAPDH.

**Fig 1:**
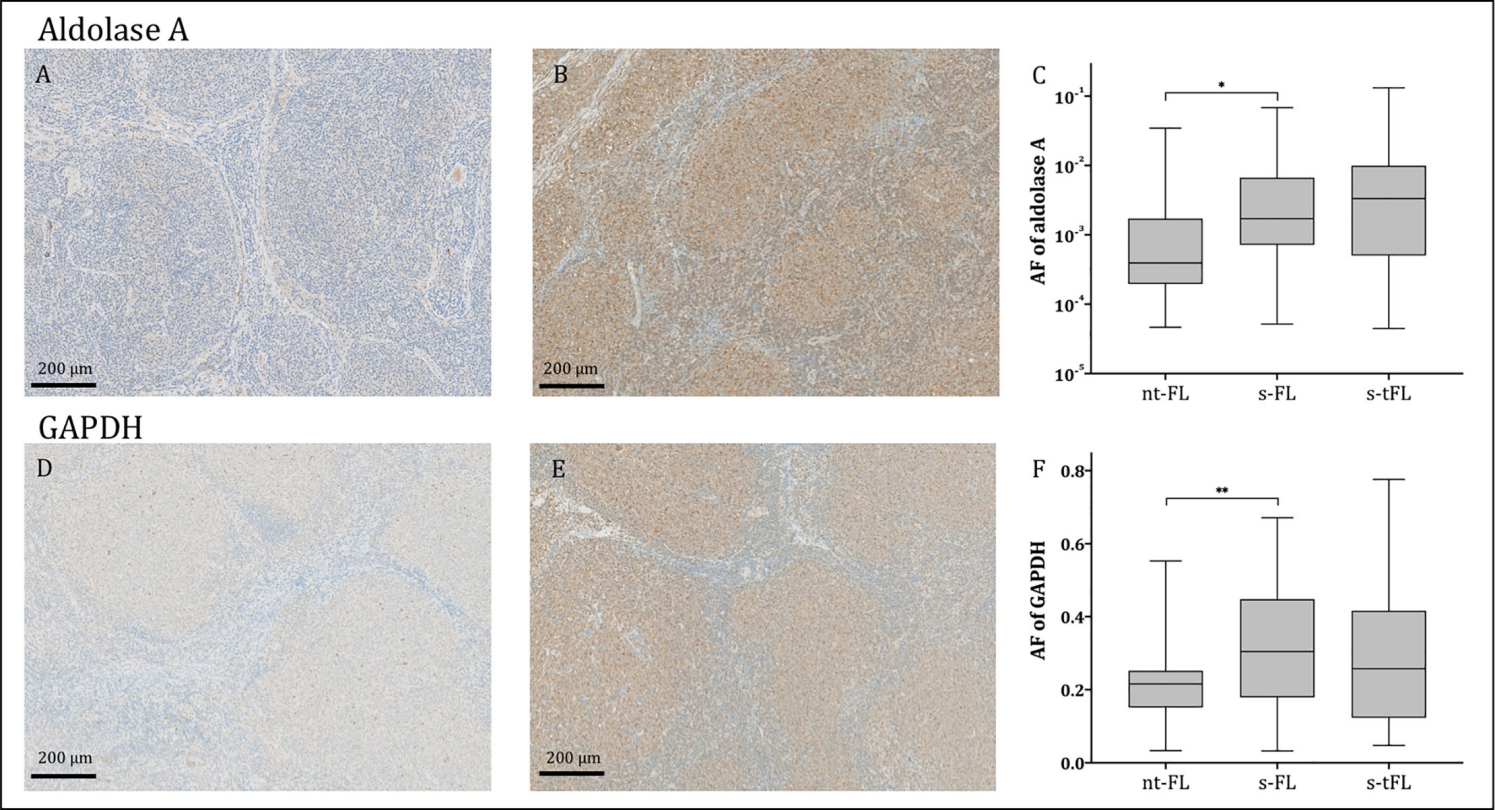
Predictive biomarker expression levels. Representative images of tumor tissues stained for aldolase A (A-B) and GAPDH (D-E). A and D show tumor tissue from patients with no subsequent transformation (nt-FL), while images B and E show tumor tissue from the initial FL diagnosis, from a patient with subsequent transformation (s-FL). Area fractions of strong intensity aldolase A staining (C) and GAPDH (F) were significantly higher in FL diagnostic samples of patients with subsequent transformation (s-FL) compared with patients without (nt-FL); *p<0.001, **p<0.01. Two patients were not eligible for GAPDH IHC analysis and thus 39 nt-FL patients remained in this group (F). No significant difference in aldolase A and GAPDH expression was observed between FL diagnosis (s-FL samples) and time of transformation (s-tFL samples) (C and F). AF, area fraction; immunohistochemistry; FL, follicular lymphoma; FLIPI, follicular lymphoma international prognostic index; GAPDH, glyceraldehyde-3-phosphate dehydrogenase; HT, histological transformation; LDH, lactate dehydrogenase; PFS, progression free survival; TFS, transformation free survival.

At time of initial FL diagnosis, patients with subsequent HT (s-FL) had significantly higher levels of aldolase A and GAPDH expression compared with patients with no subsequent transformation (nt-FL), (p<0.001 and p<0.01; Fig 1). No significant difference in aldolase A and GAPDH expression was observed between FL diagnosis (s-FL samples) and time of transformation (s-tFL samples).

All clinico-pathological features that differed significantly between patients with (s-FL) or without (nt-FL) HT, including Ann Arbor stage, FLIPI score, LDH elevation, and bone marrow involvement (Table 1), and additional clinically relevant features, including sex, age, and FL grade were assessed for correlation with aldolase A and GAPDH expression levels. None of these parameters was significantly correlated with either aldolase A or GAPDH expression levels.

**Fig 2:**
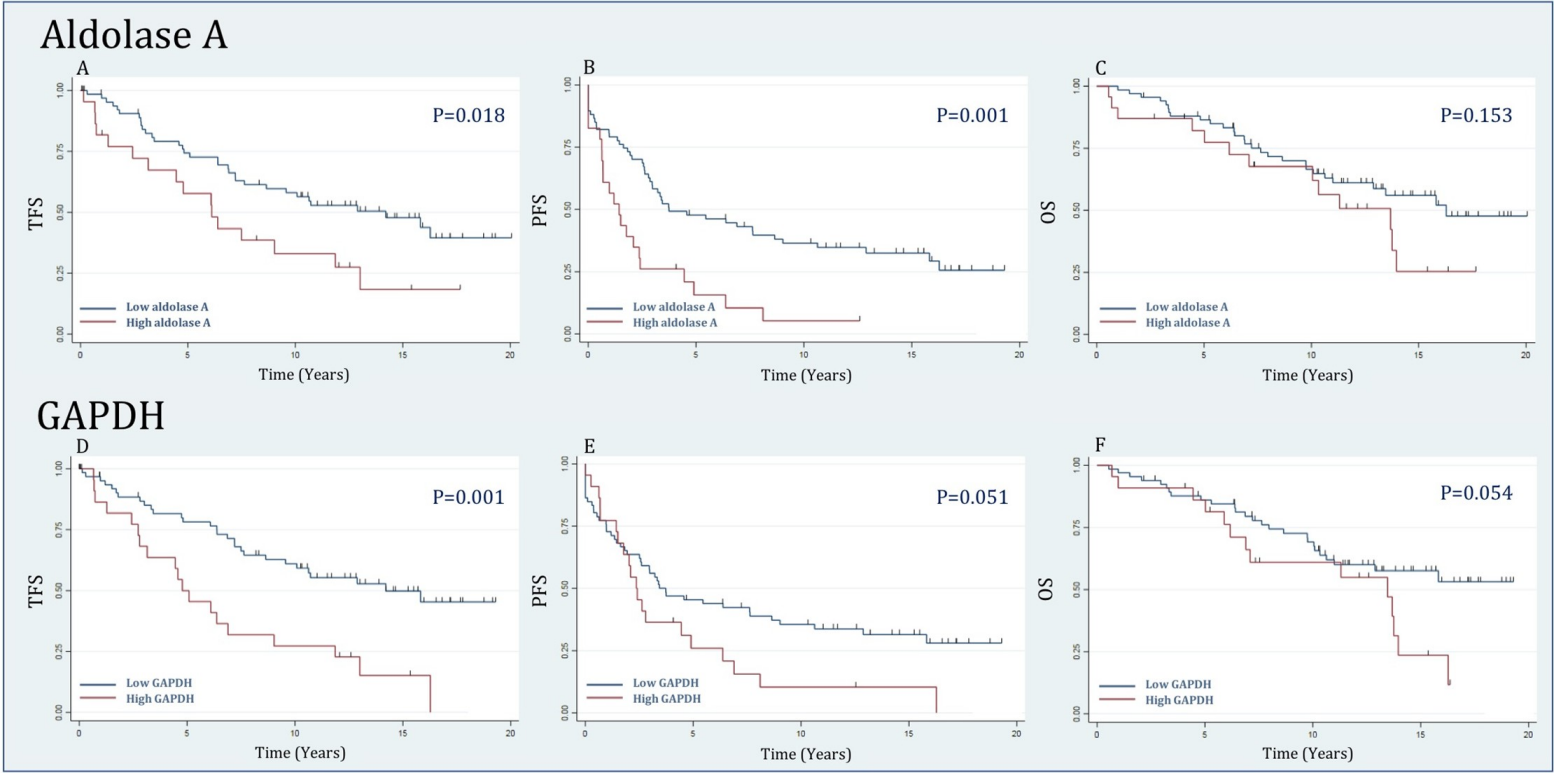
Outcome analysis. High levels of aldolase A expression were found to be associated with a significantly shorter transformation free survival (TFS) (p = 0.018) (A) and shorter progression free survival (PFS) (p = 0.001) (B). Furthermore, high levels of GAPDH expression were found to be associated with a significantly shorter TFS (p = 0.001) (D) and a trend towards shorter PFS (p = 0.051) (E). Overall survival was not significantly associated with either aldolase A and GAPDH expression levels (C and F). The low aldolase A expression group (n = 67) included 44 nt-FL and 23 s-FL patients, whereas the high aldolase A expression group (n = 23) consisted of 5 nt-FL and 18 s-FL (Table 1). The low GAPDH expression group (n = 66) included 44 nt-FL and 22 s-FL patients whereas the high GAPDH expression group (n = 22) consisted of 5 nt-FL and 17 s-FL (Table 1). FL, follicular lymphoma; GAPDH, glyceraldehyde-3-phosphate dehydrogenase; OS, overall survival; PFS, progression free survival; TFS, transformation free survival.

High levels of aldolase A and GAPDH expression at time of FL diagnosis were found to be associated with a significantly shorter TFS (p = 0.018 and p = 0.001) and PFS for aldolase A (p = 0.001), but this was only trending for GAPDH (p = 0.051; Fig 2). OS did not show a significant association with expression levels of either aldolase A or GAPDH.

## Discussion

We show that aldolase A and GAPDH immunohistochemical expression levels are increased at primary FL diagnosis in tumors from patients who subsequently experience histological transformation, and that high expression of these enzymes is associated with a significantly shorter TFS. Importantly, we used whole tissue sections, thus taking non-neoplastic cells of the tumor microenvironment into account in the biomarker analysis. We believe, that the transformation promoting effects that appear to be associated with expression of these two glycolytic enzymes may be attributed, not just to the tumor cells, but to the underlying FL tumor biology as a whole, representing a tumor phenotype or microenvironment that supports the process of HT. It is well-known, that malignant cell growth requires high levels of adenosine triphosphate (ATP) production, for which glucose is the main catabolite. The metabolism of glucose produces pyruvate, which can enter one of two pathways. Pyruvate is either metabolized into lactate in the cytoplasm through glycolysis, or enters the tricarboxylic acid (TCA) cycle via conversion into Acetyl-CoA, resulting in oxidative phosphorylation [24–26]. Whether the process of HT is dependent on either of these pathways require further investigation. However, the high expression levels of the two glycolytic enzymes found in this study could suggest a general increase of the glycolytic pathway in FL patients in risk of subsequent transformation. This is supported by the impact on TFS for both aldolase A and GAPDH protein expression at time of FL diagnosis in that high levels of aldolase A and GAPDH correlates with significantly shorter TFS in our study. These results await further investigation and validation in larger and independent cohorts.

Metabolic heterogeneity of different tumor cell subsets and adjacent non-neoplastic tissue cells, has previously been shown to promote metabolic coupling, where cells exchange metabolites based on their preferred metabolic state in order to optimize their metabolic capacity [24,27]. The metabolic state of FL, as well as tFL clones, is not well-explored [26]. However, a study of metabolic pathways in DLBCL by Gooptu *et al*., identified a shift towards ATP production through oxidative phosphorylation in DLBCL tumor cells, compared with surrounding non-neoplastic cells [27]. Additionally, upregulation of lactate exporters (MCT4) on non-neoplastic cells and lactate importers (MCT1) on tumor cells was found [27]. Thus, the ATP production of neoplastic cells was attributed to not only metabolic turnover of glucose into pyruvate followed by oxidative phosphorylation by tumor cells, but also to glycolysis in non-neoplastic cells resulting in lactate production, subsequently transported into tumor cells for lactate disposal and ATP production via oxidative phosphorylation [24,27]. It could be speculated that aldolase A and GAPDH both play a role in promoting a highly glycolytically active FL tumor microenvironment that supports the growth of transformed tFL subclones, possibly depending on mitochondrial respiration, via metabolic coupling allowing sufficient metabolic demands to be met.

Regardless of the preferred metabolic state of transformed FL cells, the notion of a generally increased glycolysis in transformation prone FL tumors, is in itself rather interesting. Enhanced glycolysis, resulting in excessive lactate production, has long been a central hallmark of cancer, described as the Warburg effect. However, this idea has been puzzling, given our understanding of effective metabolism, since glycolysis is a much less ATP effective metabolic pathway than mitochondrial respiration under aerobic conditions [28]. Advances in cancer metabolism research have given new clues as to why this metabolic pathway pertains in cancer, providing reason to believe that lactate is not simply a waste product at the end of the road of the Warburg effect, but that it is also highly carcinogenic and has cancer promoting effects related to all of the hallmarks of cancer [28].

Identification of optimal cut-off values for high/low expression and if the evaluation should be based on all staining intensities in the tissue section or more restricted to only high intensities of aldolase A and GAPDH is a prerequisite for the prospective use of these putative predictive markers of transformation in the routine risk assessment of the FL patients. This is the second study, we have reported, in which markers initially identified in our 2D-PAGE/MS-based proteomics analysis [11], have shown to be of predictive potential when assessed by immunohistochemistry on FFPE tissue in this patient cohort. The first of these markers was vimentin which we also found to be predictive of HT risk, based on intratumoral expression levels [22]. It is evident from our data that the expression levels of these biomarkers vary within each group (nt-FL, s-FL). Thus, clinical risk stratification could include these markers in combination with both clinical parameters as well as other putative predictive markers including mutations and genomic aberrations, with the aim of identifying those FL patients at high risk of subsequent transformation.

Given the markedly poor clinical behavior and outcome associated with HT, the early detection of reliable predictors for HT would be highly valuable in the clinical setting, allowing attempts to be made at pre-emptive therapeutic intervention strategies [6]. Moreover, a better understanding of the biology behind the clonal evolution leading to HT would aid the identification of critical targets and thereby optimize therapeutic choices.
